# Mental Fatigue Modulates Dynamic Adaptation to Perceptual Demand in Speeded Detection

**DOI:** 10.1371/journal.pone.0028399

**Published:** 2011-12-01

**Authors:** Robert Langner, Simon B. Eickhoff, Michael B. Steinborn

**Affiliations:** 1 Department of Psychiatry, Psychotherapy and Psychosomatics, Medical School, RWTH Aachen University, Aachen, Germany; 2 Neuropsychology Section, Department of Neurology, Medical School, RWTH Aachen University, Aachen, Germany; 3 Institute of Neuroscience and Medicine (INM-2), Research Centre Jülich, Jülich, Germany; 4 Institute of Clinical Neuroscience and Medical Psychology, Heinrich Heine University, Düsseldorf, Germany; 5 Evolutionary Cognition, Psychological Institute, University of Tübingen, Tübingen, Germany; 6 Perception and Cognition, Psychological Institute, University of Tübingen, Tübingen, Germany; University of British Columbia, Canada

## Abstract

When stimulus intensity in simple reaction-time tasks randomly varies across trials, detection speed usually improves after a low-intensity trial. With auditory stimuli, this improvement was often found to be asymmetric, being greater on current low-intensity trials. Our study investigated (1) whether asymmetric sequential intensity adaptation also occurs with visual stimuli; (2) whether these adjustments reflect decision-criterion shifts or, rather, a modulation of perceptual sensitivity; and (3) how sequential intensity adaptation and its underlying mechanisms are affected by mental fatigue induced through prolonged performance. In a continuous speeded detection task with randomly alternating high- and low-intensity visual stimuli, the reaction-time benefit after low-intensity trials was greater on subsequent low- than high-intensity trials. This asymmetry, however, only developed with time on task (TOT). Signal-detection analyses showed that the decision criterion transiently became more liberal after a low-intensity trial, whereas observer sensitivity increased when the preceding and current stimulus were of equal intensity. TOT-induced mental fatigue only affected sensitivity, which dropped more on low- than on high-intensity trials. This differential fatigue-related sensitivity decrease selectively enhanced the impact of criterion down-shifts on low-intensity trials, revealing how the interplay of two perceptual mechanisms and their modulation by fatigue combine to produce the observed overall pattern of asymmetric performance adjustments to varying visual intensity in continuous speeded detection. Our results have implications for similar patterns of sequential demand adaptation in other cognitive domains as well as for real-world prolonged detection performance.

## Introduction

Detection latency in simple reaction-time (RT) tasks regularly decreases with increasing stimulus intensity or size [1], [2]. When, however, stimuli of different intensities are unpredictably mixed within a block of trials, RT has been shown to be additionally modulated by sequential intensity dependencies. Specifically, detection speed was found to improve on the trial immediately following a low-intensity stimulus, regardless of the intensity on the current trial ([3]–[7]; see Ref. [8] for a review]. Furthermore, using auditory stimuli, John [3], Kellas [4], and Murray [5] reported that this sequential modulation was stronger with lower than higher stimulus intensities on the current trial. The only study on sequential intensity effects that used visual stimuli [7], however, did not find this overadditive interaction between preceding and current stimulus intensity. The author himself suggested that the absent interaction might be related to not having manipulated stimulus size. He argued that visual stimuli, as opposed to auditory ones, have the second dimension of stimulus size, which may interact with brightness in ways that might necessitate their combined manipulation to achieve interactions with other variables on RT (for related evidence, see Refs. [9], [10]). Our first question, therefore, was whether asymmetric (overadditive) sequence effects of stimulus intensity occur in a visual simple-RT task when brightness covaries with size, that is, when brighter stimuli are also larger than dim stimuli.

Different from earlier studies, we used a continuous RT task without explicit warning signals prior to each imperative stimulus. Interspersed warning signals might undermine the validity of sequential intensity effects by producing sequential modulations of their own [11]–[13]. In fact, the use of such warning signals might have contributed to the failure to observe significant sequential intensity effects in two early studies [12], [14].

Traditionally, asymmetric sequential intensity effects were interpreted within the framework of Grice's [15] variable-criterion model. This model holds that sensory input (i.e. perceptual evidence) elicits neural activation that accrues with a given rate. Once the accumulating evidence reaches a preset detection criterion, a decision about the presence of a given stimulus is made, and a response can be given. In the context of sequential intensity effects it was argued that after a low-intensity signal, participants adopt a lower detection criterion on the next trial, speeding up the response to a forthcoming signal regardless of its own intensity [8], [16]. Since perceptual evidence for low-intensity stimuli is thought to accumulate more slowly than evidence for high-intensity stimuli [15], the benefit (i.e., the RT reduction) from a criterion down-shift is greater for low- than for high-intensity stimuli. This hypothesis of intertrial criterion shifts, however, may have been premature, since it was only inferred from sequential modulations of RT, without having been tested directly. Therefore, an alternative explanation of these sequence effects based on trial-to-trial changes of perceptual sensitivity (instead of the response criterion) cannot be ruled out. This alternative perspective could assume that a perceptually demanding (i.e. low-intensity) stimulus enhances perceptual sensitivity on the subsequent trial, mediated by an attentional tuning of perceptual processors.

Both accounts make similar predictions about RT and errors of omission (i.e., shorter RT and less omissions after a low-intensity trial), but they differ in their assumptions about the underlying mechanisms: the classic variable-criterion view assumes that a preceding low-intensity trial lowers the threshold for the amount of accumulating sensory evidence required for detection, whereas the variable-sensitivity view assumes that a preceding low-intensity trial steepens the evidence input function. Signal detection theory (SDT; [17], [18]) provides a means to directly test and, thus, distinguish both accounts. SDT assumes that the perceptual input channel is noisy and that sometimes noise alone is sufficient to reach the detection criterion and elicit a response. These invalid (or “premature”) responses are typically called false alarms. From the number of false alarms and the number of valid responses (“hits”), independent measures of observer bias (i.e. the decision criterion) and observer sensitivity (i.e. detection ability) can be derived to directly test the prediction of intertrial criterion shifts versus sensitivity changes after stimuli of different intensity.

Although the application of SDT to simple-RT tasks is a non-standard approach, since no “genuine” catch trials are included, we think that two features of our paradigm enabled the extension of the SDT logic to our data: (i) we used a continuous RT task, without warning signals that indicate the start of a new trial, and (ii) we used non-aging interstimulus intervals [19], [20]. During such intervals, the objective conditional probability of the next stimulus occurrence (i.e. the hazard rate) does not change with elapsing time. Together, these features resulted in a situation where participants could expect the occurrence of a stimulus at (almost) any moment, which effectively turned interstimulus intervals into catch trial–like epochs. Accordingly, we considered any premature response a false alarm.

In a final step, we examined how mental fatigue, induced by increasing time on task (TOT), affects sequential intensity adaptation. Fatigue from prolonged performance has been repeatedly shown to impair simple-RT performance [21]–[23], and on several occasions, significant performance decrements in tasks requiring stimulus detection or discrimination were observed within periods as short as 10 min [24], [25]. Using SDT measures, many studies have shown that mental fatigue occurring with prolonged continuous stimulus discrimination leads to a reduction of perceptual sensitivity [25], [26]. This deterioration of sensitivity, which plays a major role in the widely known vigilance decrement, is thought to result from a fatigue-induced depletion of attentional resources ([25], [27]–[29]; see also Ref. [23]). On the other hand, various studies on vigilance also reported significant increases in the decision criterion with TOT ([30], [31]; see Ref. [32] for a review). We, therefore, reasoned that if TOT-induced mental fatigue affects observer bias, sensitivity or both, the asymmetric pattern of sequential intensity effects on RT performance might *change* or, alternatively, might even only *appear* over time.

In sum, this study pursued three goals: First, we tested whether randomly varying visual target intensity (i.e. bright and large vs. dim and small squares) in a continuous simple-RT task induces asymmetric sequential performance adjustments. Second, we aimed to elucidate the nature of these sequential modulations (i.e. criterion shifts vs. sensitivity changes) by analysing SDT-derived measures of observer bias and sensitivity. Third, we examined how mental fatigue influences the pattern of sequential intensity adaptation.

## Methods

### Ethics Statement

The study was approved by the local ethics committee of the RWTH Aachen University Hospital. All participants gave their written informed consent prior to entering the study.

### Participants

The sample comprised 37 (16 female) healthy volunteers, aged 19 to 30 (*M* = 23.3, *SD* = 2.8) years, who were paid for their participation. All but one participant reported to be right-handed, and all had normal or corrected-to-normal vision. Self-reports indicated that nobody had slept unusually little the night before or had consumed substantial amounts of alcohol the day before or unusual amounts of nicotine or caffeine on the day of testing.

### Task and Procedure

**Figure 1:**
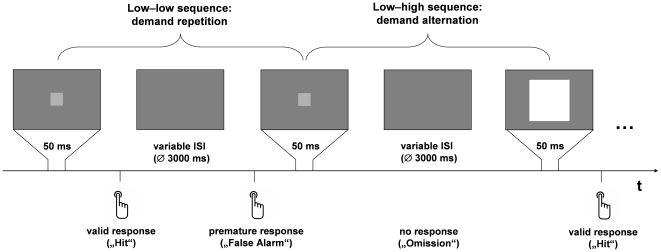
Trial structure and response types. Participants were to detect – as fast as possible – low- and high-intensity squares presented in random order. In the actual task, the low-intensity target square was smaller and less bright relative to the background; it was enhanced here for display purposes. The variable, non-aging interstimulus interval (ISI) was sampled from an exponential distribution. Detection responses were considered valid when reaction time was between 120 and 550 ms.

Sitting approximately 60 cm in front of a computer screen in a dimly lit and quiet room, participants were to respond as fast as possible to a square, appearing at the centre of the screen, by lifting the index finger of their dominant hand from an optical response button. The stimuli comprised large, high-intensity squares (19.85° visual angle; 90 cd/m^2^) and small, low-intensity squares (0.96° visual angle; 17 cd/m^2^), presented in random order for 50 ms each on a dark-grey (16 cd/m^2^) background (cf. Figure 1). The duration of the interstimulus interval varied randomly and was sampled from an exponential distribution with a mean of 900 ms plus a constant period of 2100 ms. The task was presented via a standard personal computer using Presentation 10.0 (Neurobehavioral Systems Inc., USA); it lasted 25 min in total.

The Short Questionnaire for Current Strain (KAB; [33]) was administered directly before and after the task to assess subjective perceptions of strain and fatigue. This self-report measure comprises eight pairs of adjectives on 6-point Likert-type rating scales describing opposite endpoints of different strain dimensions (e.g. stressed vs. relaxed; languid vs. fresh).

### Data Analysis

The trials of the first minute were considered practice and excluded from analysis. Performance measures comprised individual mean RT (based on valid responses with an RT between 120 and 550 ms) and omission rate (i.e. percentage of missed responses, including responses more than 550 ms after stimulus onset). Button presses up to 119 ms after stimulus onset were not considered a reaction to the stimulus but a premature response to noise fluctuations (i.e. a false alarm). The 550-ms upper RT cut-off was chosen in line with previous work on speeded detection using the same task [23]. By using this seemingly low upper bound we aimed to restrict valid responses to “speeded hits” (i.e. responses in the typical speed range of simple-RT tasks), excluding any trial on which detection could be assumed to be slowed by extraneous sources that lead to reduced task engagement (cf. Refs. [34], [35]). In comparison with applying a less strict upper RT threshold (i.e. between 800 and 1000 ms), however, the number of slow responses additionally excluded was negligible.

Omission rate (OR) was rather low and, therefore, arcsine-transformed for inferential statistics. This procedure, commonly applied to normalize skewed distributions of proportions, transformed the relative frequency of errors of omission, *p*, as follows: *p*' = 2 × arcsin 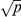. Transformed values were only employed for statistical testing; for descriptive statistics, original values were used.

SDT measures for observer criterion (*c*) and sensitivity (*d*') were based on *z*-transformed relative frequencies of valid responses (hits) and premature responses (false alarms; see Table S1 for descriptive statistics). For analysis, a trial was considered to start immediately after the response to the previous stimulus. Thus, a variable expectancy period (usually termed “foreperiod”), ranging from previous response to current stimulus onset, constituted the first section of each trial. Any button press during this response–stimulus interval (and up to 119 ms after current stimulus onset; cf. above) was considered “premature” and, therefore, a false alarm (cf. Figure 1). In case there was no valid response to the previous stimulus, any premature button press on the current trial was categorized as false alarm only when at least 2000 ms since the previous trial's stimulus onset had elapsed. This prolonged interval was chosen to minimize the risk of confusing a late response to the previous stimulus with a false alarm on the current trial. The number of such instances, however, was negligible; leaving them out of the analysis did not change the results. Standard SDT measures were calculated as follows: *c* = –0.5 × [*z*(hits) + *z*(false alarms)]; *d*' = *z*(hits) – *z*(false alarms). Data sets with no false alarms or omissions, respectively, were corrected by a standard procedure: zero values were replaced by 1/(2 × n), with n being the maximum number of false alarms or omissions (i.e. the number of trials).

For analysing TOT effects, performance measures were separately calculated for six consecutive 4-min time bins. RT, OR, *c* and *d*' were analysed by 6 × 2 × 2 repeated-measures analyses of variance (ANOVAs) with factors TOT (6 time bins), intensity on the previous trial (INT_n−1_: high vs. low), and intensity on the current trial (INT_n_: high vs. low). Since fatigue effects can start occurring rather early during the task (i.e., after about 5 min; cf. Ref. [25]) and their onset cannot be determined unequivocally, we examined TOT effects by conservative a-priori defined Helmert contrasts that compared the first time bin against the rest. The significance threshold was set at *p*<.05. Perceived task-induced mental fatigue was assessed by comparing KAB total scores from before and after the session by means of a paired *t*-test.

## Results

**Figure 2:**
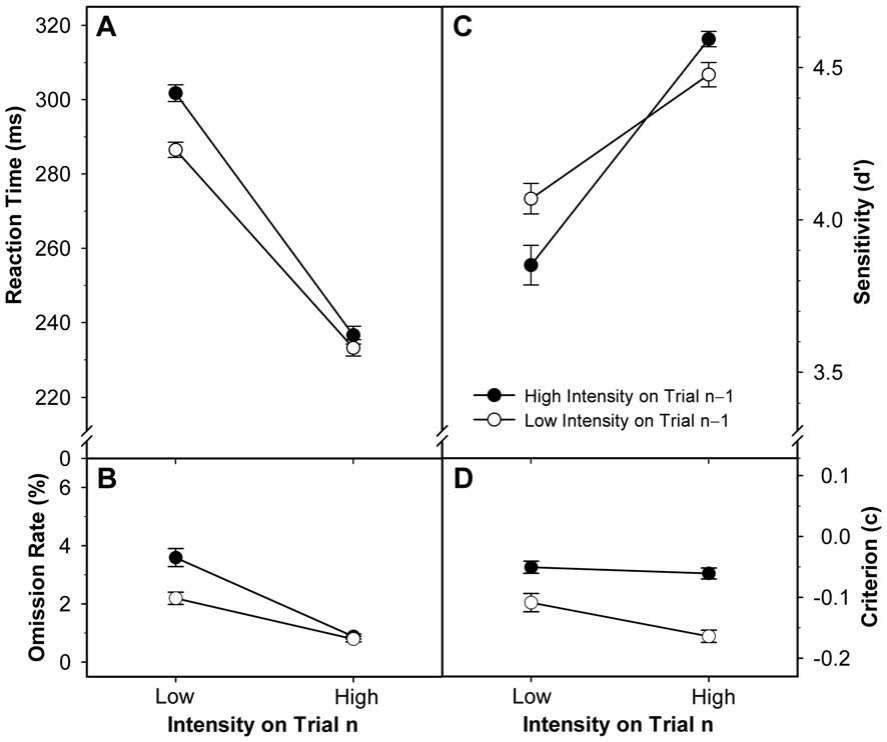
Performance as a function of stimulus intensity on the current and previous trial. Panel A: reaction time; panel B: percentage of missed responses; panel C: observer sensitivity; panel D: observer bias. Each measure results from averaging across the entire session. Error bars represent standard errors of the mean; connecting lines between data points were added for illustrative purposes.

As expected, RT was significantly shorter on high- than low-intensity trials as well as following a low- versus high-intensity trial (Figure 2A and Table S1; see Table 1 for statistics). Both effects interacted significantly, indicating that the speed-up following low-intensity trials was more pronounced when the current stimulus was of low intensity, too. The interaction, however, was ordinal, corroborating a global sequential intensity effect that reflects generally faster responses after perceptually demanding low-intensity trials.

**Table 1:** Results of the Analyses of Variance of Reaction Time and Percentage of Missed Responses (Omission Rate).

Source	Reaction Time			Omission Rate		
	*F*	*p*	*η_p_^2^*	*F*	*p*	*η_p_^2^*
INT_n_	1605.48	<.001	0.98	35.37	<.001	0.47
INT_n-1_	34.40	<.001	0.49	11.42	.002	0.24
TOT	13.29	<.001	0.27	17.12	<.001	0.32
INT_n_ × INT_n−1_	33.26	<.001	0.48	7.20	.011	0.17
INT_n_ × TOT	5.23	.028	0.13	5.05	.031	0.12
INT_n−1_ × TOT	<0.01	.557	0.01	0.55	.462	0.02
INT_n_ × INT_n−1_ × TOT	7.26	.011	0.17	1.55	.222	0.04

*Note*. Degrees of freedom: 1, 36. *η_p_^2^* = partial eta^2^ (effect size); INT_n_/INT_n−1_ = stimulus intensity (high vs. low) on the current/previous trial; TOT = time on task (Helmert contrast between the first and the remaining five 4-min time bins).

**Figure 3:**
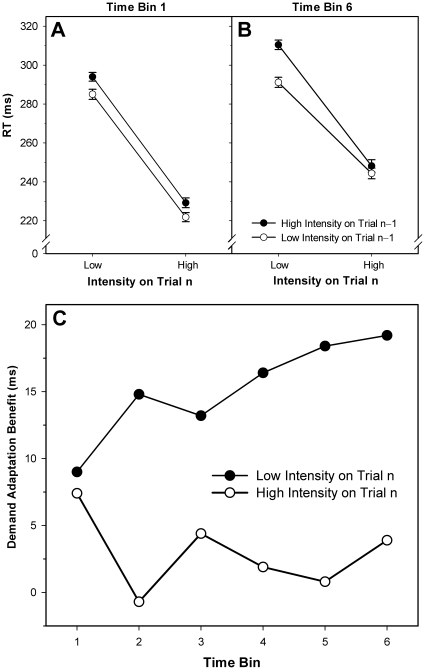
Reaction time as a function of time on task. Panels A and B: Reaction time separately averaged for the first (A) and last (B) time bins as a function of stimulus intensity on the current and previous trial. Panel C: Difference in mean reaction time between trials preceded by a low- versus a high-intensity trial (“demand adaptation benefit”) for each time bin, separately shown for current low- and high-intensity trials. Error bars represent standard errors of the mean; connecting lines between data points were added for illustrative purposes.

Furthermore, the analysis yielded a main effect of TOT, since RT increased significantly over time (see Figures 3A,B). The significant INT_n_ × TOT interaction was further qualified by a hybrid INT_n_ × INT_n−1_ × TOT interaction, revealing that for high-intensity trials, the speed gain following a low-intensity trial slightly decreased over time, whereas for low-intensity trials, the gain increased (see Figure 3C). In other words, the observed INT_n_ × INT_n−1_ interaction (i.e. the asymmetry of the perceptual demand adaptation benefit) only emerged with TOT. As alluded to above, effects of TOT were tested by a-priori defined Helmert contrasts; however, similar effects also emerged in the “full” ANOVA, contrasting RT values for each of the six time bins (see Table S2).

Main effects on OR (i.e. errors of omission) mirrored RT effects (Figure 2B and Table S1; see Table 1 for statistics), making it unlikely that increases in RT resulted from shifts of the speed–accuracy trade-off towards greater accuracy. Similar to RT, there also was an ordinal INT_n_ × INT_n−1_ interaction effect on OR, showing that the decrease in omission errors after low-intensity trials was significantly stronger on current low- than high-intensity trials. The significant INT_n_ × TOT interaction indicated a stronger OR increase over time for low-intensity trials. There was no other significant interaction.

The only effect on the decision criterion was a significant influence of previous stimulus intensity: *c* became lower (i.e. more liberal) after a low-intensity trial (Figure 2D; see Table 2 for statistics). As expected, observer sensitivity *d*' (Figure 2C; see Table 2 for statistics) was significantly better on current high- than low-intensity trials, but the preceding trial's intensity produced no significant main effect on *d*'. There was, however, a hybrid INT_n_ × INT_n−1_ interaction, revealing that the INT_n_ main effect on *d*' (i.e. the difference in sensitivity between high- and low-intensity trials) was significantly smaller after low- than high-intensity trials. When comparing intensity repetitions with alternations, this INT_n_ × INT_n−1_ interaction corresponds to the intensity-repetition main effect and indicates a significant repetition benefit (i.e. increased *d*' on the current trial when following a trial with equal stimulus intensity). Thus, whereas the detection criterion generally became more liberal after a perceptually demanding (i.e. low-intensity) stimulus, observer sensitivity did not show this effect. Rather, it was generally enhanced after intensity repetitions, as compared to alternations.

**Table 2:** Results of the Analyses of Variance of Decision Criterion (c) and Observer Sensitivity (d').

Source	Criterion (c)			Sensitivity (d')		
	*F*	*p*	*η_p_^2^*	*F*	*p*	*η_p_^2^*
INT_n_	3.05	.089	0.08	17.85	<.001	0.33
INT_n−1_	15.29	<.001	0.30	0.54	.465	0.02
TOT	0.34	.562	0.01	15.26	<.001	0.30
INT_n_ × INT_n−1_	1.01	.321	0.03	8.73	.005	0.20
INT_n_ × TOT	2.01	.165	0.05	6.70	.014	0.16
INT_n−1_ × TOT	0.21	.650	0.01	0.17	.687	0.01
INT_n_ × INT_n−1_ × TOT	0.66	.422	0.02	2.45	.126	0.06

*Note*. Degrees of freedom: 1, 36. *η_p_^2^*  =  partial eta^2^ (effect size); INT_n_/INT_n−1_  =  stimulus intensity (high vs. low) on the current/previous trial; TOT  =  time on task (Helmert contrast between the first and the remaining five 4-min time bins).

**Figure 4:**
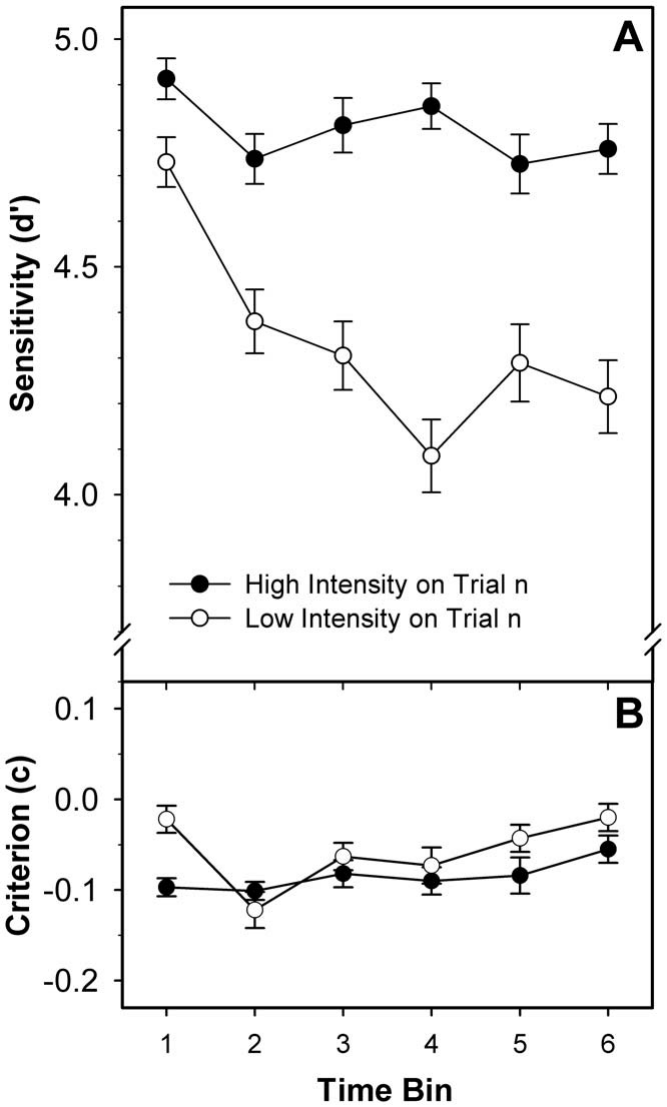
Development of observer sensitivity (panel A) and observer bias (panel B) over time. Results are separately shown for current low- and high-intensity trials. Error bars represent standard errors of the mean; connecting lines between data points were added for illustrative purposes.

The criterion *c* did not interact with TOT, but *d*' significantly decreased over time (cf. Table 2). This main effect was further qualified by a significant INT_n_ × TOT interaction, revealing that the time-related decline of *d*' was stronger for current low- than high-intensity stimuli (cf. Figure 4).

Corresponding to the performance decrement with TOT, the KAB pre-task score (*M*  =  18.9, *SD*  =  2.8) was significantly lower than the post-task score [*M*  =  22.4, *SD*  =  6.3, *t*(36)  =  –3.5, *p*  =  .001]. This increase in subjective strain indicates that our task induced mental fatigue.

## Discussion

In a visual simple-RT task with imperative stimuli of variable intensity (i.e., easy- vs. hard-to-detect brightness–size combinations), we found intensity-related sequential performance adjustments. Specifically, low intensity on a given trial entailed improved detection speed on the subsequent trial. These findings agree well with previous reports [3]–[7]. Further, we found an (overadditive) interaction between current and preceding stimulus intensity. That is, responses on low-intensity trials were substantially faster when preceded by a low-intensity trial than when preceded by a high-intensity trial, while responses on high-intensity trials were only slightly faster when preceded by a low-versus a high-intensity trial (cf. Figure 2A). Sequence effects similar to RT were found for OR: the percentage of missed (including very slow) responses was lower after low-intensity trials, with a stronger effect on current low-intensity trials.

### Mechanisms Underlying Asymmetric Sequential Intensity Adaptation

Our result contrasts with the only previous study on sequential intensity effects using visual stimuli [7] but accords with earlier findings on auditory stimuli [3]–[5]. As alluded to above, observing this asymmetric sequential adaptation effect might be related to our covarying stimulus brightness and size in the same direction, which enhanced the difference in stimulus intensity and, thus, the sequential impact. Importantly, this interaction was ordinal, that is, an RT improvement – albeit of different magnitude – occurred after a low-intensity trial on both current low- and high-intensity trials. Thus, performance somewhat improved even on intensity alternation trials (i.e. low–high intensity sequences), ruling out an explanation of the improvement based on intensity-repetition benefits. Such an explanation would simultaneously predict alternation costs, i.e., an RT *increase* on low–high intensity sequences, as compared to high–high intensity sequences, which obviously was not observed. This is not to say, however, that repetition/alternation effects did not play a role at all: despite having been outweighed by demand-related adaptation (i.e. the global performance improvement after a low-intensity trial), they may have contributed to the asymmetry of the adaptation. We will return to this issue in more detail shortly.

Applying SDT, we tested two accounts of the mechanisms proposed to underlie the observed sequential intensity adaptation: intertrial shifts of the detection criterion versus changes in detection sensitivity. We found that after low-intensity trials, the criterion *c* on the subsequent trial was lowered, independent of the subsequent trial's intensity. That is, high perceptual demand on the previous trial made the observer more liberal, in line with the classic interpretation of sequential intensity effects on RT as resulting from intertrial shifts of the criterion ([3], [5], [7]; see Ref. [8] for a review]. Nevertheless, we also found effects of the previous trial's intensity on observer sensitivity (*d'*), which did, however, depend on the current trial's intensity. Specifically, after low-intensity trials, sensitivity *increased* on subsequent low-intensity trials but *decreased* on subsequent high-intensity trials. This hybrid interaction effect is equivalent to a global increase in sensitivity on intensity repetitions (i.e. high–high and low–low intensity sequences), as compared to intensity alternations. Thus, in contrast to the observer's criterion, sensitivity showed clear repetition benefits and costs.

**Figure 5:**
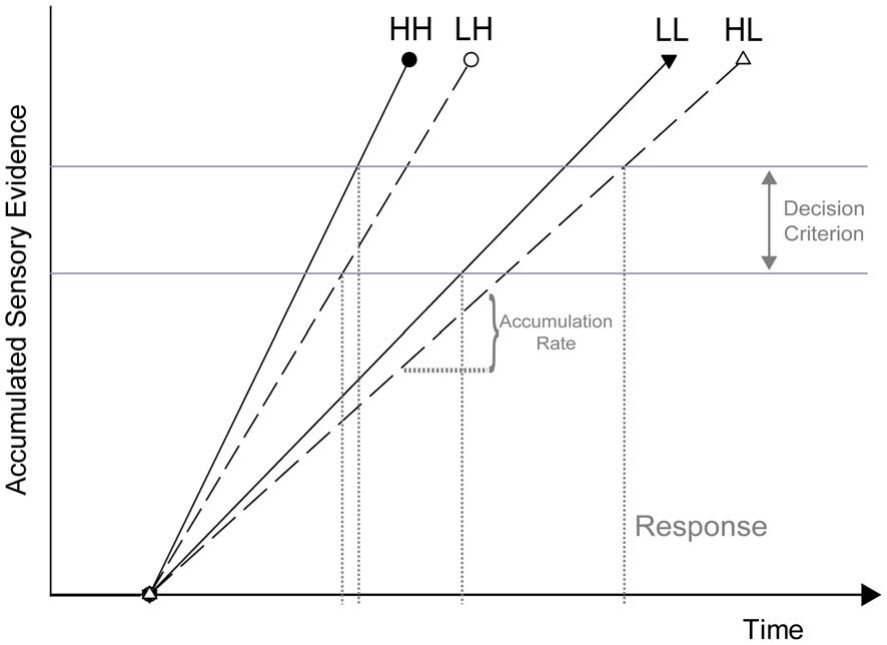
Illustration of the results (averaged across time) in the context of the variable-criterion model [**15**]. Sensory evidence accrues faster upon presentation of a high-intensity (H) stimulus, relative to a low-intensity (L) one. This initial difference is further modulated by stimulus intensity on the preceding trial: when intensity is repeated on the current trial (HH or LL trial sequence), the rate of evidence accumulation is increased, relative to intensity alternations (HL or LH trial sequence). At the same time, the decision criterion, which the accruing evidence needs to reach for a response to be emitted, is lowered after a preceding low-intensity trial and raised after a high-intensity trial. The interplay of these intertrial criterion shifts and the effects of current and previous stimulus intensity on the evidence accumulation rate (i.e. observer sensitivity) result in the observed pattern of response times.

Figure 5 visualizes the pattern of results in the framework of Grice's [15] variable-criterion model. In this model, the rate of sensory evidence accumulation increases with observer sensitivity. According to our results, evidence accrual is faster on trial repetitions than on trial alternations (i.e., it is faster on low–low than on high–low intensity sequences as well as on high–high than on low–high intensity sequences). This, in turn, suggests that the greater sequential RT benefit on low- than high-intensity trials not only derives from a lower basic rate of evidence accumulation on low-intensity trials. Rather, this initial difference appears to be further enhanced by a beneficial intensity-repetition effect on sensitivity: on *low*-intensity trials, the already large RT gain from a given criterion down-shift after a preceding high-intensity trial is *further enlarged* by a flattening of the evidence input function with an intensity *alternation*; thus, the RT difference between high–low and low–low intensity sequences is *enhanced*. In contrast, on *high*-intensity trials, the already small RT gain from a given criterion down-shift after a preceding high-intensity trial is *further diminished* by a steepening of the evidence input function with an intensity *repetition*; thus, the RT difference between high–high and low–high intensity sequences is *reduced*. A caveat regarding these inferences from our SDT analyses should be noted here, though: Our RT task departed from the typical situation signal-detection analyses are applied to, in that hit rate was high, and false-alarm rate was low. This might have lowered the reliability of the parameter estimates, which should be taken into account when interpreting our SDT results.

In sum, RT benefits after trials with high perceptual demand (i.e. low stimulus intensity) appear to be largely mediated by criterion down-shifts; however, the resulting initial difference in these benefits between low- and high-intensity trials appears to be further enhanced by sequential sensitivity modulations (i.e. repetition gains and alternation costs). These conclusions corroborate the classic explanation of sequential intensity adaptation by intertrial criterion shifts (cf. Refs. [8], [16]) but they also show that this explanation should be extended to include the beneficial effect of intensity repetitions on evidence accumulation rate.

With regard to the mechanisms behind dynamic sensitivity changes, it was previously shown that perceptual sensitivity strongly depends on the allocation of attention [36], [37]. For instance, Reynolds, Pasternak and Desimone [38] reported that spatial attention enhanced effective stimulus strength, along with increasing activity in brain areas involved in visual perception. Likewise, improvements in perceptual discrimination were found with spatial or temporal manipulations that enhanced the allocation of attention [39]–[43]. Previous research suggested several mechanisms through which a sensitivity increase by attention may be mediated, such as signal enhancement [40] or a higher rate of information processing [44]. Although our data do not allow us to pinpoint the exact mechanism(s) at work here, the above findings suggest that the significant sensitivity benefits on trial repetitions are mediated through a predictive tuning of perceptual processors by stimulus-driven attention.

### Modulation of Sequential Intensity Adaptation by Mental Fatigue

Our fatigue manipulation appeared to have worked well: self-reported strain levels (as measured by the KAB) were higher after than before the task, and performance demonstrated the global decrement typically associated with growing mental fatigue [21]–[24]. Since simple-RT tasks are hardly susceptible to practice [45], they are ideally suited to capture fatigue-related changes in attentiveness ensuing over time. It is also for this reason that these tasks are frequently used for (repeated) assessments of alertness in chronobiological, neuropsychological or sleep-deprivation research [46]–[48]. We did not find, however, a global increase in the sequential intensity effect on performance with TOT-induced fatigue. Instead, we found that the speed-up following low-intensity trials was differentially affected by fatigue, depending on the current perceptual demand: on high-intensity trials, the benefit remained about stable (or, if anything, slightly decreased) over time, whereas on low-intensity trials, it grew substantially. Put another way, the difference in the RT benefit after perceptual demand observed between subsequent high- and low-demand trials increased with fatigue (cf. Figure 3C). In conclusion, the overall interaction pattern observed was not stable over time: at the beginning (i.e. in time bin 1), there was no interaction at all; it only appeared with TOT-induced mental fatigue.

The finding that the stronger impact of previous intensity on current low- as compared to high-intensity trials only develops with increasing TOT strongly suggests that the two-way interaction between previous- and current-trial intensity might not only depend on stable “structural” factors (i.e. the difference in intensity), as previously assumed. The question arises whether in earlier studies reporting this interaction [3]–[5] a similar dependence on TOT could have been found but just went unnoticed. However it may be, this dependence argues for a crucial role of “energetic” factors in producing the overadditivity of the interaction between previous- and current-trial intensity, at least in visual simple-RT tasks.

To elucidate the underlying mechanisms, we analysed the impact of TOT-induced fatigue on criterion shifts and sensitivity changes. We did not find any changes in the criterion with TOT, but sensitivity declined over time, with the decline being more pronounced on low- than high-intensity trials (cf. Figure 4). In terms of Grice's [15] model, this TOT-related sensitivity decline slows sensory evidence accumulation. Since the slowing is asymmetric, the impact on RT of a given criterion down-shift after a preceding low-intensity trial (the amount of which remained stable over time) is selectively enhanced on subsequent low-intensity trials. Therefore, notably, the observed asymmetry of sequential intensity effects appears to be largely brought about by a differential time-related decline in observer sensitivity. It should be noted, though, that this need not necessarily apply to previous findings with auditory stimuli: there, initial intensity differences might have been large enough to produce sufficiently strong sensitivity differences from the start. We conclude that – at least for visual stimuli – the interaction pattern between preceding and current stimulus intensity is not a purely perceptual effect but depends on a modulation by mental fatigue.

Finding sensitivity decreases rather than criterion increases over time agrees with frequent observations in studies on the vigilance decrement (for a review, see Ref. [26]). The lack of TOT effects on the criterion is presumably due to the lack of non-target trials in our simple-RT task. Vigilance studies typically employ discrimination tasks with many more non-target than target events. There, criterion increases over time have been typically interpreted as an adjustment (i.e. reduction) of initial subjective estimates of target probability to the actual, mostly rather low, target rate [32]. Since target probability was 100% in our task, the lack of such expectancy changes with TOT is not surprising.

The observed steeper sensitivity decline for perceptually demanding (vs. non-demanding) stimuli also replicates findings with vigilance tasks [25]. The sensitivity decrement with TOT has frequently been ascribed to a depletion of attentional resources with increasing task-induced mental fatigue [28], [29]. Dwindling attentional resources may also explain the asymmetry of the decline, since perceptually demanding stimuli require more top-down attention to be processed efficiently and, consequently, their detectability should suffer more from resource depletion than that of highly salient stimuli [23], [28].

However, for interpreting the interplay between the level of top-down attention and the level of (bottom-up) stimulus salience, another feature of our results is informative: At the beginning of the task, sensitivity differences between low- and high-intensity trials were hardly present; they only developed over time. This pattern replicates previous findings with a continuous discrimination task using little degraded and highly degraded visual stimuli [25]. The pattern suggests that the to-be-expected sensitivity difference between high and low intensities is initially compensated, presumably by top-down attention, and, further, that this compensation vanishes with TOT. This notion is in line with a view of mental fatigue as an imbalance between energetic costs and perceived rewards of continued task performance [49]. Accordingly, to avoid this imbalance in our task, participants might have adjusted their energetic costs (“perceived effort”) to the perceived (rather moderate) benefit from continued performance by choosing a less effortful strategy, i.e. reducing compensatory top-down attention.

This view also strengthens the validity of the asymmetric overall interaction pattern, since the time-related change of this pattern towards asymmetry would then essentially indicate a transition to a more stimulus-driven, “natural” performance pattern, which is, over time, increasingly less “distorted” by effortful (over)compensation. It remains for future studies to test whether a more *symmetric* sequential intensity adaptation can be observed with stimuli whose salience difference is smaller (requiring less effortful compensation on low-intensity trials) or whether an even more *asymmetric* sequential intensity adaptation can be induced with stronger fatigue manipulations. On a more general note, our results may be taken as a reminder to take energetic factors such as fatigue or motivation into account when theorizing about speeded performance.

### Relation to Other Task Domains

Intriguingly, the pattern of sequential intensity effects on detection latency resembles sequential effects observed when experiencing cognitive conflict (i.e., conflict adaptation). Originally demonstrated in a task in which irrelevant flanking letters interfered with processing a central target letter by evoking conflict between competing response tendencies [50], performance on conflict trials was often shown to improve when the immediately preceding trial also required conflict resolution, compared to when it did not. This effect presumably arises from adapting cognitive-control parameters following the registration of conflict ([51]; see Ref. [52] for a review). Challenging this control-based explanation, it was shown that some of these sequential effects may be accounted for by repetition priming [53] or, more generally, by an episodic memory retrieval of the stimulus–response association formed on the previous trial [54]. Recent studies, however, indicate that control-based effects co-exist with associative memory effects [52].

This dual-process view on sequential effects in conflict tasks corresponds to our finding of two separable mechanisms (i.e. demand-triggered criterion shifts and repetition-related sensitivity changes) that appear to jointly produce the asymmetric interaction pattern reported above. Apart from this correspondence, however, there also is a notable difference: Experiencing conflict usually induces a performance *decrease* on subsequent non-conflict (i.e. low-demand) trials [51], whereas in our task, low-intensity (i.e. high-demand) trials induced a slight detection *improvement* on subsequent high-intensity (i.e. low-demand) trials. This difference to typical findings in conflict tasks might result from paradigm-specific factors, about which we can presently only speculate. In conflict paradigms, for instance, alternation costs might be greater than, and therefore outweigh, demand adaptation effects. Any difference that remains after appropriately controlling for alternation effects in conflict tasks might arise from different mechanisms mediating dynamic demand adaptation in the two tasks. Thus, in a typical conflict paradigm like the flanker task, for example, conflict is thought to enhance cognitive control on the subsequent trial, presumably by improving the selectivity of spatial attention. This, in turn, reduces the beneficial impact of congruent flanker stimuli. In effect, this preparatory attentional modulation might lead to increased sensitivity in target processing [40], whereas in our task, high perceptual demand appears to lead to a generalized bias for detection. In conclusion, it would be interesting to examine whether in tasks tapping cognitive control, the effects of previous control demand and repetition benefits can also be dissociated by a selective association with bias or sensitivity, and, if so, how these associations might differ from perceptual demand adaptation in speeded detection tasks like ours.

This question also applies to tasks in other domains outside the realm of cognitive conflict, where similar patterns of sequential performance adjustments to variable task demands were observed. For instance, conflict-unrelated dynamic performance adjustments were found to be elicited by differential working-memory demands [55]. Fischer, Dreisbach and Goschke [56] reported that number comparisons were solved faster after a difficult trial (i.e., small numerical distance) than after an easy one (i.e., great numerical distance). In a related study [57], the difficulty of categorizing number words was manipulated by degrading the words on half the trials, resulting in a similar interaction as reported here: the difference in RT between low- and high-demand trials was greater after a low- than after a high-demand trial. In contrast to our results, however, this interaction was not ordinal, as there was no global performance gain after high-demand trials. The slight RT increase on difficult–easy, relative to easy–easy, sequences reported by the authors might reflect a stronger impact of demand alternation costs than that observed in our task. When assuming that, as in our task, demand alternation negatively affects sensitivity, it might well be that in a number categorization task (which is substantially more complex than simple detection by requiring stimulus identification, number processing and response selection), the alternation-driven slowing of evidence accumulation outweighs the beneficial down-shift of the decision criterion after high demand. The reasoning that an alternation-related *sensitivity* reduction lies at the heart of the RT increase on difficult–easy sequences is further supported by the – at least numerical – increase of this performance drop over time, which agrees with our finding that TOT-induced fatigue primarily reduces observer sensitivity. Thus, fatigue-enhanced alternation costs (e.g. arising from reduced compensatory attention) may have contributed to the time-related change in the pattern of sequential demand adaptation reported in that study.

Examining how criterion shifts and sensitivity changes interact could also inform the “search image” literature in behavioural ecology, which assumes stimulus-specific tuning of perception during search for prey [58]. Analyses of sequential dependencies revealed that target detection in birds improved after a preceding target had been detected successfully [59]. This was interpreted as resulting from a reinforcement of the search image (i.e. a selective perceptual tuning) on the previous trial. Since no signal-detection analyses were employed, it remains open whether sequential criterion changes were involved besides any putative repetition-based changes in sensitivity.

### Future Directions

Apart from the generalizability of our findings across other task domains, several other questions emerged from our study that future research should address. For instance, the effect of different brightness–size combinations should be examined systematically. Similarly, future studies should independently vary stimulus intensity and stimulus type (e.g. form) to disentangle (general) demand-repetition from (specific) item-repetition effects. Future studies should also consider using quasi-continuous or at least multi-level variations of stimulus intensity to enable examining sequential intensity and TOT effects on parameters of the presumably sigmoid function that relates current stimulus intensity to detection probability and speed. Furthermore, systematic investigation is needed of the effects of different stimulus modalities (including their mixed presentation) as well as different and potentially stronger energetic manipulations (e.g. sleep deprivation, circadian variation, or pharmacological interventions such as caffeine). Finally, future studies could examine interactions between sequential intensity effects and other manipulations that potentially influence perceptual sensitivity, such as the probability of specific intensity levels [5] or sequential fluctuations of exogenous temporal attention with variable interstimulus intervals.

Our observation that detection performance is not affected uniformly by fatigue but modulated by sequential adjustments to stimulus intensity also has implications for real-world settings that involve a fatigue-inducing continuous monitoring of variably salient items, such as airport luggage inspection. Apparently, mental fatigue most strongly impairs detecting a perceptually demanding, non-salient item in the wake of a non-demanding, salient one. Based on these findings, an investigation of analogous effects in safety-relevant real-life settings may be warranted.

### Conclusion

Unpredictable trial-to-trial variation in auditory stimulus intensity in speeded detection tasks was previously found to elicit asymmetric sequential performance adjustments. Here we show that similar adjustments occur with visual stimuli: detection performance improved after a perceptually demanding (i.e. low-intensity) stimulus but did more so when the current stimulus was demanding, too. Signal-detection analyses suggested that an interplay of demand-triggered down-shifts of the detection criterion and repetition-related sensitivity increases jointly produced the observed performance pattern. Notably, the asymmetry in sequential intensity adaptation only emerged with time on task, arguing for a profound role of energetic factors such as mental fatigue in producing the overall interaction. As a result, the variable-criterion model [15], traditionally used to explain sequential adjustments to variable stimulus intensity, should be amended by including benefits for observer sensitivity from trial repetitions and asymmetric sensitivity modulations by TOT-induced mental fatigue. The occurrence of similar adaptation patterns across various cognitive domains (e.g. conflict adaptation) invites the question for common and distinct underlying perceptual and decisional mechanisms. From the pervasiveness of such “on-line” adjustments it would appear that they are a useful mechanism for successfully processing the signals of our natural environment, which often poses unpredictably varying perceptual and attentional demands (e.g. signals with variable salience).

## Supporting Information

Table S1 **Performance Measures as a Function of Trial Type and Time on Task, Separately Averaged for Each of the Six Consecutive Time Bins.**
(PDF)

Table S2 **Results of the Analyses of Variance of Reaction Time and Percentage of Missed Responses (Omission Rate) for the Effects of Time on Task Using Six Separate Time Bins.**
(PDF)
