# Supplementary material for: Mental Fatigue Modulates Dynamic Adaptation to Perceptual Demand in Speeded Detection
Source: PLoS One. 2011 Dec 1;6(12):e28399. doi: 10.1371/journal.pone.0028399 (PMC3228758; doi:10.1371/journal.pone.0028399)
Supplement: Table S1 — Performance Measures as a Function of Trial Type and Time on Task, Separately Averaged for Each of the Six Consecutive Time Bins. (PDF) [file pone.0028399.s001.pdf]

Supplementary Material

Table S1
Performance Measures as a Function of Trial Type and Time on Task, Separately Averaged for Each of the Six Consecutive Time Bins

Trial Type	Reaction Time (ms)		Omissions (%)		False Alarms (%)	
	M	*SD*	*M*	*SD*	*M*	*SD*
Time Bin 1						
High–High sequence	229	30	0.5	1.7	1.0	2.9
Low–High sequence	222	29	0.7	2.7	2.9	5.0
High–Low sequence	294	31	2.6	4.5	2.4	3.5
Low–Low sequence	285	31	0.5	1.9	1.3	2.8
Time Bin 2						
High–High sequence	229	26	0.4	1.6	1.5	2.7
Low–High sequence	230	30	1.0	2.1	3.1	4.1
High–Low sequence	297	32	2.0	3.8	3.5	4.2
Low–Low sequence	282	26	2.0	3.2	3.5	3.8
Time Bin 3						
High–High sequence	237	30	0.7	2.2	1.4	3.0
Low–High sequence	232	28	0.9	2.2	3.1	5.8
High–Low sequence	300	29	3.9	6.8	3.9	6.1
Low–Low sequence	287	26	2.1	4.6	3.9	5.1
Time Bin 4						
High–High sequence	237	36	0.7	2.3	2.0	4.5
Low–High sequence	235	30	0.7	2.2	2.1	3.3
High–Low sequence	301	26	4.6	6.2	4.9	6.1
Low–Low sequence	284	24	2.4	3.1	4.7	5.5
Time Bin 5						
High–High sequence	239	34	1.7	3.9	2.6	4.6
Low–High sequence	238	33	0.6	1.8	2.0	3.0
High–Low sequence	311	34	4.2	6.5	4.0	6.2
Low–Low sequence	292	32	2.6	4.6	4.0	5.6

Time Bin 6						
High–High sequence	248	39	1.7	3.2	1.5	3.2
Low–High sequence	244	33	0.8	3.0	2.8	4.5
High–Low sequence	310	30	4.3	5.1	5.0	6.2
Low–Low sequence	291	32	3.2	6.6	3.2	5.3

Note. High = high intensity (i.e. bright and large stimulus); Low = low intensity (i.e. dim and small stimulus)
