# Supplementary material for: Mental Fatigue Modulates Dynamic Adaptation to Perceptual Demand in Speeded Detection
Source: PLoS One. 2011 Dec 1;6(12):e28399. doi: 10.1371/journal.pone.0028399 (PMC3228758; doi:10.1371/journal.pone.0028399)
Supplement: Table S2 — Results of the Analyses of Variance of Reaction Time and Percentage of Missed Responses (Omission Rate) for the Effects of Time on Task Using Six Separate Time Bins. (PDF) [file pone.0028399.s002.pdf]

Supplementary Material

Table S2

Results of the Analyses of Variance of Reaction Time and Percentage of Missed Responses (Omission Rate) for the Effects of Time on Task Using Six Separate Time Bins

Source	Reaction Time			Omission Rate		
	F	p	η_p^2	F	p	η_p^2
TOT	9.99	<.001	0.22	3.21	.017	0.08
INT_n × TOT	2.98	.013	0.08	1.48	.198	0.04
INT_{n-1} × TOT	0.51	.731	0.01	1.73	.129	0.05
INT_n × INT_{n-1} × TOT	2.30	.047	0.06	0.82	.538	0.02

Note. Degrees of freedom: 5, 180. η_p^2 = partial eta² (effect size); INT_n/INT_{n-1} = stimulus intensity (high vs. low) on the current/previous trial; TOT = time on task (six 4-min time bins).

Whenever appropriate, the Greenhouse–Geisser correction was used to compensate for violations of the sphericity assumption.
